# Supplementary material for: Biphasic Response of Astrocytic Brain-Derived Neurotrophic Factor Expression following Corticosterone Stimulation
Source: Biomolecules. 2022 Sep 18;12(9):1322. doi: 10.3390/biom12091322 (PMC9496348; doi:10.3390/biom12091322)
Supplement: Supplementary file 1 [file biomolecules-12-01322-s001.zip › biomolecules-1752464-supplementary.pdf]

Supplementary Material

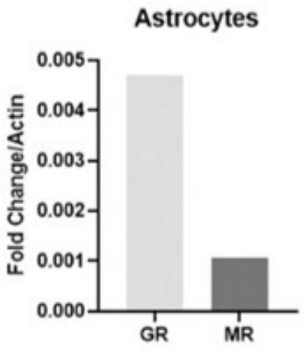

Figure S1. Supporting data Relative expression levels between GR and MR on astrocytes. The mRNA levels are expressed as the Fold Change expression of each receptor compared to the expression levels of the housekeeping gene Actin. Astrocytes express GR almost 4 times higher compared to MR.

Gene ID	Forward	Reverse	Product size
Bdnf	GGGTCACAGCGGCAGATAAA	GCAGCCTTCCTTGGTGTAAC	149
Ntrk2	CCTGATGGCCAAGAACGAGT	GGCGTGGTCCAGTCTTCATA	129
Actin	GGAGATTACTGCTCTGGCTC	GGACTCATCGTACTCCTGCT	151
Nr3c1	TTGGGGGCTATGAACTTCGC	CTGGTCTCATTCCAGGGCTTG	70
Nr3c2	GAGCCGTGGAAGGACAACA	GGCTTGGAGGCATTTCTGGA	113
Per1	GGAGTTGGCCCTCTGATGTC	TGCTGGAAAGTCACTGGAGC	113

Table S1. Mouse primer sequences.
